# Porous Silk Fibroin Microspheres Sustainably Releasing Bioactive Basic Fibroblast Growth Factor

**DOI:** 10.3390/ma11081280

**Published:** 2018-07-25

**Authors:** Jing Qu, Lu Wang, Longxing Niu, Jiaming Lin, Qian Huang, Xuefeng Jiang, Mingzhong Li

**Affiliations:** 1 National Engineering Laboratory for Modern Silk, College of Textile and Clothing Engineering, Soochow University, No. 199 Ren’ai Road, Industrial Park, Suzhou 215123, China; jqu@suda.edu.cn (J.Q.); wdl02011@126.com (L.W.); niulongxing@126.com (L.N.); 20165215002@stu.suda.edu.cn (J.L.); 20164215010@stu.suda.edu.cn (Q.H.); jiangxfsuda@163.com (X.J.); 2 Nantong Textile and Silk Industrial Technology Research Institute, No. 266 New Century Avenue, Nantong 226000, China

**Keywords:** silk fibroin, microspheres, controlled release, basic fibroblast growth factor

## Abstract

Basic fibroblast growth factor (bFGF) plays a significant role in stimulating cell proliferation. It remains a challenge in the field of biomaterials to develop a carrier with the capacity of continuously releasing bioactive bFGF. In this study, porous bFGF-loaded silk fibroin (SF) microspheres, with inside-out channels, were fabricated by high-voltage electrostatic differentiation, and followed by lyophilization. The embedded bFGF exhibited a slow release mode for over 13 days without suffering burst release. SEM observations showed that incubated L929 cells could fully spread and produce collagen-like fibrous matrix on the surface of SF microspheres. CLSM observations and the results of cell viability assay indicated that bFGF-loaded microspheres could significantly promote cell proliferation during five to nine days of culture, compared to bFGF-unloaded microspheres. This reveals that the bFGF released from SF microspheres retained obvious bioactivity to stimulate cell growth. Such microspheres sustainably releasing bioactive bFGF might be applied to massive cell culture and tissue engineering as a matrix directly, or after being combined with three-dimensional scaffolds.

## 1. Introduction

Basic fibroblast growth factor (bFGF) has been shown to play key roles in stimulating neovascularization [1], wound repair [2] and tissue regeneration [3], as well as promoting the reconstruction of cartilage and bone [4]. bFGF performs its biological functions by enhancing cellular proliferation and self-refreshing in the growth of embryonic stem cells [5], neural stem cells [6], vascular endothelial cells [7] and fibroblasts [8]. Despite these therapeutic effects, it is still a challenge to completely realize the biological effects of exogenous bFGF. This is due to limitations such as short half-life in vivo, and loss of activity in physiological environment—owing to the combination with plasma proteins, factors and certain types of ions; destruction due to pH and temperature; enzymatic hydrolysis; and cellular uptake [2,9,10]. Therefore, developing a carrier that can sustainably release bioactive exogenous bFGF may have vital significance in massive cell culture, tissue engineering and in situ tissue regeneration.

Synthetic polymers, commonly used as bFGF, release carriers covered poly(lactic-*co*-glycolic acid) (PLGA), poly(l-lactic acid) (PLLA) and polycaprolactone (PCL). These polymers containing ester bonds are biodegradable, and the biodegradation rate can be controlled by changing the degree of crystallinity or block copolymerization [11,12,13,14]. Nevertheless, the primary drawback is that the acidic degradation products may cause tissue inflammation in the degradation process [15,16,17]. Furthermore, the innate hydrophobicity of these macromolecules leads to a decrease in surface wettability, which is unfavorable for maintaining original molecular conformation and bioactivity of loaded drugs [13,18,19]. The natural polymers usually used as carriers for bFGF are collagen [20] and gelatin [21]. Both materials have the common disadvantages of limited mechanical strength, and fast degradation without adequate time to support cell proliferation and maintain extracellular matrix deposition [22].

Silk fibroin (SF) has attracted considerable attentions as drug sustained-release carriers attributed to its cytocompatibility and controllable biodegradability. Previous research has shown that bFGF combined to SF membrane by using nontoxic chondroitin sulfate could be sustainably released for 30 days, and such SF membrane with bFGF could not only sustain L929 cell growth but also promote cell differentiation [23]. Moreover, the released bFGF from silk scaffold coated with ultrafine electrospun bFGF-PLGA fibers could stimulate initial proliferation and subsequent differentiation of the BMSCs [24]. Furthermore, as the carriers of VEGF [25] or IGF-I [26], SF could continuously release bioactive growth factors over periodic time. All these indicate that silk fibroin is suitable for the carrier material of drug release.

SF can be processed into nanostructured materials such as nanofibers [27] and nanoparticles [28] or other materials formats including microspheres, films, hydrogels and three-dimensional scaffolds for its superior mechanical and physical properties [29]. In addition with large amounts of amino and carboxyl groups in the side chains of SF, biological and chemical modifications can be performed to engraft special function groups on the SF: Imparting unique properties to SF-based materials [30]. Electrostatic interactions and unique secondary structure might play a role in maintaining the stability of bioactive molecules, such as enzymes or polypeptide drugs loaded in SF materials [31]. The chemical composition, secondary structure and assembly in nanoscale of SF make this natural protein an attractive candidate for the stabilization of bioactive drugs such as proteins, peptides, and nucleic acids over extended periods [32].

In the present study, the bFGF-loaded porous SF microspheres were contaminant-free fabricated by differentiating bFGF/SF aqueous solution into droplets under a high-voltage electrostatic field and followed by lyophilization. We hypothesized that the interactions between silk fibroin and bFGF would be beneficial for stabilizing the bioactivity of bFGF, and the inside-out interstices produced during the lyophilization process in the interior of SF microspheres could provide channels for the release of bFGF. Such particles could stimulate cell proliferation within several days by releasing bioactive bFGF sustainably and slowly. We investigated the in vitro release of bFGF from SF microspheres by means of ELISA assay. We also inoculated and cultured mouse lung fibroblast cells L929 on the surface of bFGF-loaded SF microspheres to evaluate the bioactivity of released bFGF.

## 2. Results

### 2.1. Characteristics of SF Microspheres

**Figure 1:**
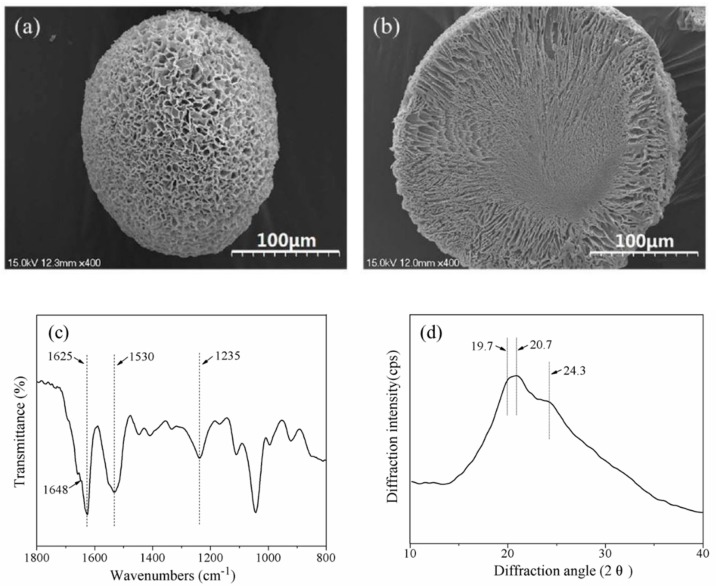
(**a**) The surface SEM image; (**b**) cross section SEM image; (**c**) Fourier transform infrared (FTIR) spectrum and (**d**) X-ray diffraction curve of silk fibroin (SF) microspheres. Scale bars: 100 μm.

Scanning Electronic Microscopy (SEM) images (Figure 1a,b) of SF microspheres showed that the particle shape was spherical or ellipsoidal, and the diameters were in the range of 95 μm to 260 μm. The surface and interior of the SF microspheres were porous, with pore sizes being approximately 1.5 μm to 7.0 μm on the surface. The width of channels extending from the interior to the surface increased along toward the surface of the microsphere. The central zone of the microsphere showed a dense morphology with less pores.

Previous studies on the molecular conformation of SF with Fourier transform infrared (FTIR) revealed that *α*-form structure usually exhibited characteristic absorption at 1650–1655 cm^−1^ (amide I), 1525–1540 cm^−1^ (amide II), 1266 cm^−1^ (amide III), and 669 cm^−1^ (amide V); while *β*-sheet structure had characteristic absorption at 1620–1635 cm^−1^ (amide I), 1530 cm^−1^ (amide II), 1230–1235 cm^−1^ (amide III), and 700 cm^−1^ (amide V) [33]. From the FTIR spectrum of SF microspheres (Figure 1c), the strong absorption at 1625 cm^−1^ (amide I) and 1530 cm^−1^ (amide II) were indicative of *β*-sheet; while the medium strong peak at 1235 cm^−1^ (amide III) and the shoulder peak at 1648 cm^−1^ (amide I) were indicative of *α*-form. Both of these characteristics revealed that the SF conformations in microspheres were mainly *β*-sheet with a small amount of *α*-form structure.

X-ray diffraction (XRD) patterns of SF using Cu K*α* radiation indicated that the main diffractions of Silk I (*α*-form) were at 12.2° (d = 72.5 nm, medium strong), 19.7° (d = 45.0 nm, strong), 24.7° (d = 36.0 nm, medium) and 28.2° (d = 31.6 nm, medium), and those of Silk II (*β*-sheet) were at 9.1° (d = 97.0 nm, medium strong), 18.9° (d = 46.9 nm, medium strong), 20.7° (d = 43.0 nm, very strong) and 24.3° (d = 36.6 nm, medium strong) [34]. A major diffraction at ~20.7° and a medium strong diffraction at ~24.3° were observed in the XRD curve (Figure 1d); furthermore, a strong diffraction appeared at ~19.7°, indicating that the SF microspheres contained a higher content of *β*-sheet and a lower content of *α*-form structure, which conformed to the results of FTIR spectrum.

### 2.2. In Vitro Release of bFGF from SF Microspheres

**Figure 2:**
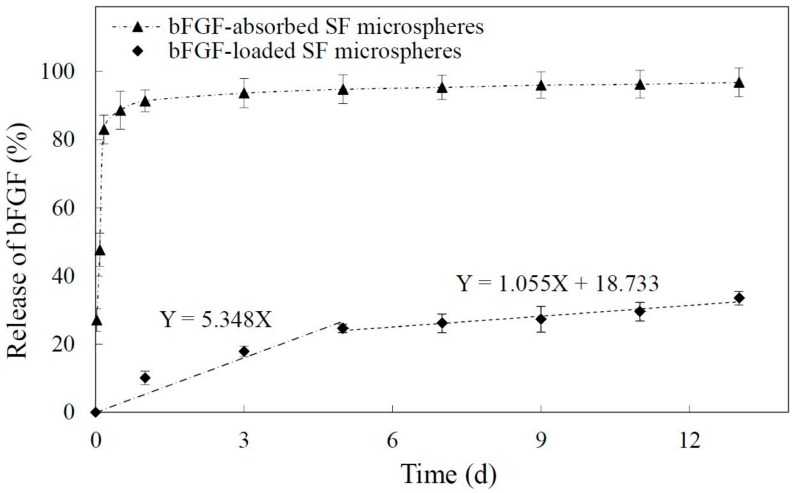
Release of basic fibroblast growth factor (bFGF) from SF microspheres in 13 days. (◆ bFGF-loaded SF microspheres showed two release stages; ▲ bFGF-absorbed SF microspheres showed initial burst release).

Figure 2 showed the release profile of bFGF from SF microspheres at 37 °C in PBS over the course of 13 days. The release of bFGF from SF microspheres experienced two phases without suffering burst release. At the early stage (1 to 5 days), the release rate was comparatively higher, and the average daily release percentage was approximately 5.3%. The regression line equation of this stage was Y = 5.348X (Y-percentage of bFGF release (%); X-time of bFGF release (day)). From 5 to 13 days, the release curve tended to be flat and steady with an average daily release percentage of 1.1%. The regression line equation of this stage was Y = 1.055X + 18.733 (Y-percentage of bFGF release (%); X-time of bFGF release (day)). According to the slopes of regression line equations from two release stages, it could be concluded that the bFGF from SF microspheres was released more gently from 5 to 13 days, indicating the bFGF reached a sustained and slow release. As a control, bFGF-absorbed SF microspheres were prepared to observe the release characteristics of bFGF-loaded SF microspheres in this study. Figure 2 shows the absorbed bFGF suffered an obvious burst release, and approximately 83% of bFGF was released in 4 h, followed by the residual ~17% slowly released from 4 h to 13 days.

### 2.3. Growth of L929 Cells on bFGF-Loaded SF Microspheres

**Figure 3:**
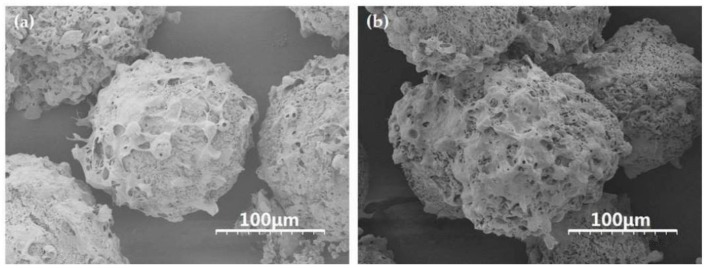
SEM images of L929 cells on the surface of (**a**) bFGF-unloaded and (**b**) bFGF-loaded SF microspheres after 5 days of culture. Scale bars: 100 μm.

L929 cells were respectively inoculated and cultured on bFGF-unloaded and bFGF-loaded SF microspheres at 37 °C in a 5% CO_2_ atmosphere. From SEM images, the cells adhered and fully spread on the surface of bFGF-unloaded/loaded microspheres tightly, and evenly with pseudopods connecting to each other to form networks after 5 days of culture (Figure 3). A mass of collagen-like fibrous matrix was formed and deposited on the surface of microspheres, especially on bFGF-loaded SF microspheres (Figure 3b). These results turned out that the SF microspheres prepared by high-voltage electrostatic differentiation and followed by lyophilization could support cell adhesion, spreading and growth on their surface, especially on the surface of bFGF-loaded SF microspheres.

**Figure 4:**
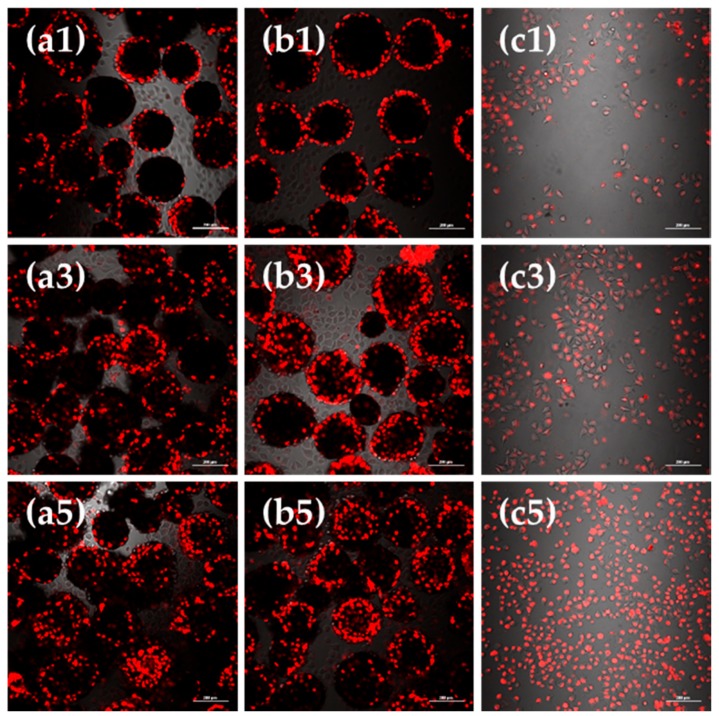
CLSM images of L929 cells cultured on (**a**) bFGF-unloaded SF microspheres; (**b**) bFGF-loaded SF microspheres; (**c**) culture plates (control) for 1, 3 and 5 days. Scale bars: 200 μm.

Observations under laser scanning confocal microscope (CLSM) showed the viable cell morphology, coherent condition, cell quantity and distribution of L929 cells cultured on bFGF-loaded SF microspheres for 1, 3 and 5 days (Figure 4). The round black regions were microspheres, while the bright and red regions represented the viable fibroblasts stained by CM-DiI fluorescent dye. Figure 4 shows that after 1 to 5 days of culture, the red fluorescent intensity of group a, b and c all enhanced, correlating to an obvious increase in number of cells. When we compared group a and b after 1 day of culture, the difference was not evident. After 3 days of culture, the fluorescence of group b was significantly stronger than group a. A similar phenomenon could be observed after 5 days of culture, reflecting that the number of cells on bFGF-loaded SF microspheres was higher than that on bFGF-unloaded ones after 3 days of culture.

**Figure 5:**
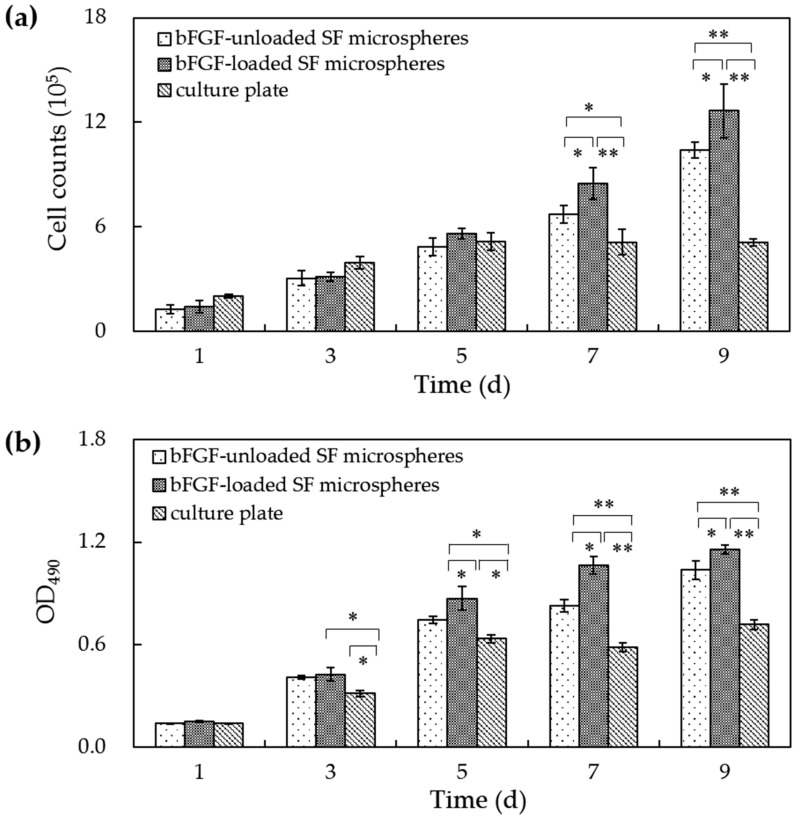
(**a**) The cell number and (**b**) cell viability on bFGF-loaded SF microspheres. (*: *p* < 0.05; **: *p* < 0.01).

Cell counts and MTT assay recorded a consistent tendency of cell numbers and cell viability on microspheres. Figure 5 shows that the cell number and cell viability on culture plates increased from 1 to 5 days of culture yet remained unchanged 5 days later. The cell numbers and cell viability of L929 cells on bFGF-loaded and bFGF-unloaded SF microspheres both rose steadily from 1 to 5 days of culture. Interestingly, 5 days later, they continued to increase until 9 days, displaying highly significant differences compared to culture plates (*p* < 0.01). Contrasting bFGF-loaded and bFGF-unloaded SF microspheres, the cell numbers and cell viability showed no distinct difference before 5 days of culture. However, from 7 to 9 days, the number and viability of L929 cells on bFGF-loaded SF microspheres were significantly higher than those on bFGF-unloaded microspheres (*p* < 0.05), suggesting the released bFGF accelerated the cell proliferation.

## 3. Discussion

This study provided a novel method of fabricating bFGF-loaded silk SF microspheres with particle size range of 95 to 260 μm, and inside-out channels by high-voltage electrostatic differentiation technique, followed by lyophilization. This method possesses mild process conditions, using aqueous SF/ bFGF solutions and low processing temperature, which is an appealing feature for the retention of bFGF bioactivity.

The surface and internal pores of microspheres were formed by the distillation of ice crystals formed in the freeze-drying process. When the differentiated droplets of SF and bFGF aqueous mixture contacted with liquid nitrogen, the outer layer of droplet was fast frozen. The cylindrical ice crystals subsequently grew from outer layer towards central zone of droplet, until it reached the glass-transition temperature of surrounding unstable phase (SF and bFGF mixture phase in droplet) [35]. After lyophilization, the channels connecting the surface and interior in the solidified droplet were shaped. The size of cylindrical ice crystals depended on the SF concentration in the surrounding unstable phase and the growth time of ice crystals. In the process of ice crystals growing from outer layer towards central zone of droplet, the surrounding unstable phase was concentrated and moved to central zone of the droplet due to the stress effects from the growth of ice crystals. Subsequently, the SF concentration in the center of droplet increased, leading to the limitation on the growth size of ice crystals. Consequently, the internal channel size of microspheres decreased from the surface to central zone after lyophilization (Figure 1b). To stabilize microspheres against water, glycerol was added into SF solution, and the mixed solution was electrostatically differentiated and lyophilized. The molecule conformation of SF in microspheres was effectively induced into *β*-sheet (Figure 1c,d), in which the SF chains were arranged in parallel with a large number of hydrogen bonds and were stable in water.

The bFGF carried a positive charge in a neutral solution, with an isoelectric point was 9.6 [36], but the SF was negatively charged, with an isoelectric point was approximately 4.2 [37]. After being added into SF solution, bFGF was combined with silk fibroin by electrostatic interaction and hydrogen bonding, dispersed in continuous SF phase, and immobilized in SF microspheres after freeze-drying. The release experiment in vitro was designed to investigate the release characteristics of loaded bFGF from SF microspheres. It was found that the bFGF was slowly released from SF microspheres for over 13 days without suffering burst release (Figure 2). The reason for this release profile could be considered to be due to that the immobilized bFGF needed to surmount not only the electrostatic adsorption and hydrogen bonding, but also the physical diffusion barrier from surrounding SF networks. In contrast, the absorbed bFGF of control group experienced an obvious burst release in 4 h because the bFGF releasing only depended on electrostatic and hydrogen bonding adsorption. Compared to bFGF-loaded PLLA scaffold [38] (exhibiting a sharp initial burst at the first day with approximately 31.0% of bFGF released from the scaffold) or bFGF-loaded PCL nanofibers [13] (showing a burst release pattern during the initial 6 h), the loaded bFGF in SF microspheres in this study avoided serious burst release. In contrast to bFGF-combined SF membrane with about 77.7% of bFGF released from the scaffold after 16 days [23], the bFGF-loaded SF microspheres in this research showed a relatively lower bFGF release proportion (about 33.5%) after 13 days. It was probably because of this that the bFGF was mainly combined to the surface of solid SF membrane in the former research. However, as the bFGF was added into SF solution in this study, after lyophilization, it was evenly immobilized to not only the surface but also the interior of SF microspheres. The bFGF inside SF microspheres was more difficult to pass through the surrounding SF networks—thus leading to a slower release.

Mouse lung fibroblast cells L929 were inoculated and cultured on bFGF-loaded SF microspheres to investigate the bioactivity of released bFGF. SEM results showed that a mass of collagen-like fibrous matrix was formed and deposited on the surface of bFGF-loaded SF microspheres after cell cultured for 5 days (Figure 3b). CLSM observations revealed that the number of cells on bFGF-loaded SF microspheres was higher than that on bFGF-unloaded ones after 3 days of culture. Further, the number and viability of L929 cells on bFGF-loaded SF microspheres were significantly higher than those on bFGF-unloaded ones after 5 to 9 days of culture (Figure 5). These results indicated that the sustainably released bFGF remained bioactive and promoted proliferation of fibroblasts effectively.

In contrast with PLGA microspheres prepared by double emulsion-solvent evaporation method [39]; gelatin particles prepared by a modified coacervation technique [36] or collagen-chitosan biofilm cross-linked by MES, EDC and NHS [40]; and porous bFGF-loaded SF microspheres were prepared by electrostatic differentiation and lyophilization in this study. The bFGF was directly mixed into SF solution, and immobilized in SF microspheres by electrostatic and hydrogen bonding adsorption. The entire process avoided the use of organic solvents and chemical crosslinking agents and maintained the bioactivity of loaded bFGF effectively. Furthermore, the porous structure and the inside-out channels of SF microspheres provided physical passages for bFGF diffusion from the interior to the surface of microspheres. These features guaranteed the continuous release of bioactive bFGF from SF microspheres.

## 4. Materials and Methods

### 4.1. Fabrication of bFGF-Loaded SF Microspheres

Silk fibroin aqueous solution was prepared as previously described [41]. Briefly, 150 g of raw silk fibers (Zhejiang the Second Silk Co. Ltd., Huzhou, China) were degummed three times in 5000 mL of 0.02 M Na_2_CO_3_ aqueous solution, and then rinsed thoroughly with deionized water. After drying in an oven at 60 °C, the extracted SF was dissolved in 9.3 M LiBr solution at 60 °C for 4 h. A 4 wt % SF solution was obtained after dialysis for 4 days in deionized water followed by filtration. The solution was diluted with deionized water to a concentration of 3 wt % and the glycerol (Sigma Aldrich Trading Co. Ltd., Shanghai, China) accounting for 30% of silk quality was added into SF solution.

**Figure 6:**
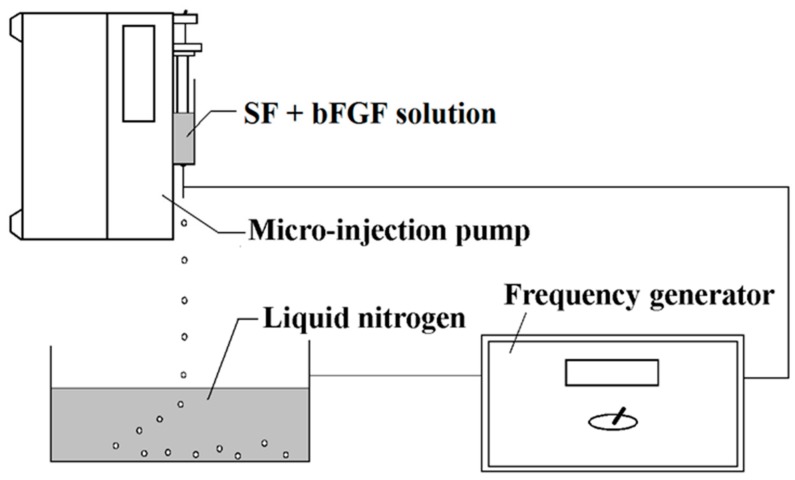
Illustration showing the experimental setup used for the preparation of SF microspheres.

The bFGF-loaded microspheres were fabricated by using high-voltage electrostatic generator (DW-P503-4ACCD, Dongwen High Voltage Power Plant, Tianjin, China) and micro-injection pump (WZS50F2, Zhejiang University Medical Instrument Co. Ltd., Hangzhou, China). As Figure 6 shows, a nozzle with diameter of 0.7 mm was linked with the syringe and the whole was fixed on the pump. The distance between the needle and the collection box was settled as 100 mm. Then, 1 μg of bFGF powder was added into 5 mL of 3 wt % SF solution (glycerol added) directly, and the mixed solution in injector was differentiated into droplets under a high-voltage electrostatic field. The produced droplets were collected and frozen in a liquid nitrogen bath. Subsequently, they were freeze-dried by a Virtis Genesis 25-LE lyophilizer for 24 h and the bFGF-loaded SF microspheres were acquired. The bFGF-unloaded SF microspheres were prepared by taking the same approach without adding bFGF powder into SF solution.

The bFGF-absorbed SF microspheres were prepared simultaneously as a control in the release experiment. The specific steps were as follows: 20 mg of bFGF-unloaded SF microspheres, prepared by previously mentioned method, were immersed in 1 mL of bFGF solution at the concentration of 250 ng/mL for 1 h at room temperature, to keep the bFGF being absorbed onto the surface and into the interior of microspheres, followed by lyophilization. The adsorption quantity of bFGF was calculated based on bFGF concentration in residual liquid measured by enzyme-linked immunosorbent assay (ELISA).

### 4.2. Morphology and Structure of Porous SF Microspheres

The SF microspheres were frozen in liquid nitrogen, and were then cut by using sharp blade to obtain the cross-section. The microspheres and the cross-section were platinum-coated, and examined morphologically by scanning electron microscopy (SEM, S-4700, Hitachi Manufacturing Co. Ltd., Tokyo, Japan). The particle size of microspheres was analyzed according to SEM images and Nano Measurer analysis software (Department of Chemistry in Fudan University, Shanghai, China). We determined the average equivalent circular diameter of 100 microspheres in total.

The freeze-dried SF microspheres were ground into powder with radii less than 40 μm, and then the samples were prepared in KBr pellets before being loaded into a FTIR instrument (Nicolet 5700, Thermo Fisher Scientific Inc., New York, NY, USA) with resolution of 4 cm^−1^, scanning range from 400 to 4000 cm^−1^, and 32 scans in total.

The freeze-dried SF microspheres were ground into powder with radii less than 40 μm. The XRD was performed by using X‘Pert-Pro MPD (PANalytical Company, Arnhem, The Netherlands) diffractometer and Cu K*α* radiation with a wavelength of 15.406 nm. The diffraction intensity curves of SF microspheres were obtained with 2θ ranging from 5° to 45°.

### 4.3. In Vitro bFGF Release from bFGF-Loaded SF Microspheres

The release profile of bFGF from SF microspheres in phosphate-buffered saline (PBS) was as follows: 20 mg of bFGF-loaded/bFGF-absorbed SF microspheres were placed in centrifugal tubes with 4 mL of PBS (pH 7.4), and were shocked in a 37 °C thermostatic water bath for release. The PBS solution in each tube containing bFGF-loaded SF microspheres was removed and transferred into a clean centrifugal tube and 4 mL of fresh PBS was supplemented at 1 days, 3 days, 5 days, 7 days, 9 days, 11 days and 13 days. As a contrast, the time points of bFGF-absorbed SF microspheres were set as 1 h, 2 h, 4 h, 12 h, 1 days, 3 days, 5 days, 7 days, 9 days, 11 days and 13 days. The amount of bFGF released from SF microspheres to PBS solution was measured by Human bFGF ELISA (Invitrogen Life Technology Co. Ltd., Carlsbad, CA, USA).

### 4.4. Bioactivity of bFGF Released from SF Microspheres

Mouse embryonic lung fibroblast cells L929 (ATCC, Manassas, VA, USA) were cultured on bFGF-loaded SF microspheres to evaluate the activity of released bFGF. Then, 3 mg of SF microspheres sterilized with *γ*-ray irradiation were steeped into culture medium containing serum for 30 min to reinforce cell adhesion, before being placed into 24-well culture plates (TCP, Corning Inc., New York, NY, USA). The L929 cells were seeded onto bFGF-unloaded SF microspheres, bFGF-loaded SF microspheres and culture plates (control) at a density of 1 × 10^5^ cells per well and afterwards incubated at 37 °C in a 5% CO_2_ atmosphere. After 5 days, cells fixed with 2.5% glutaraldehyde were incubated overnight at 4 °C. Subsequently, the fixed microsphere cultures were washed twice with phosphate-buffered saline (PBS), frozen at −80 °C for 2 h, and lyophilized for 36 h. Dry samples were platinum-coated in vacuum and examined by SEM.

The proliferation and distribution of cells on SF microspheres was observed using a confocal scanning laser microscope (CLSM, TCS-SP2, Leica Company, Weitzrah, Germany). L929 cells on bFGF-unloaded SF microspheres, bFGF-loaded SF microspheres and culture plates were labeled with CM-DiI fluorescent dye (Sibas Biotechnology Co. Ltd., Shanghai, China), then were observed by CLSM at 1, 3 and 5 days of culture.

The numbers of cells on different materials were counted using cell-count boards at 1 days, 3 days, 5 days, 7 days and 9 days. The 3-(4,5-dimethylthiazolyl-2)-2,5-diphenyltetrazolium bromide (MTT) assay was used to measure the cell viability. 200 μL of MTT dye solution (working concentration: 5 mg/mL in phosphate buffer at pH 7.4) was added into each well at 1 days, 3 days, 5 days, 7 days and 9 days. After 4 h of incubation at 37 °C and 5% CO_2_ the medium was removed, and formazan crystals were solubilized in HCL-Isopropanol overnight. The optical density (OD) of formazan was measured on a Synergy HT (BIO-TEK) microplate reader at 490 nm. Each experiment of cell counting and cell viability was performed in triplicate.

### 4.5. Statistical Analysis

All data were expressed as mean ± standard deviation (SD). Statistical comparisons were performed using one-way analysis of variance (*t*-test) and differences at *p* < 0.05 were considered statistically significant.

## 5. Conclusions

bFGF-loaded porous silk fibroin microspheres with particle size range of 95 to 260 μm and inside-out channels were prepared using high-voltage electrostatic differentiation, and followed by lyophilization. The entrapped bFGF was slowly released from SF microspheres for over 13 days without suffering burst release. Fibroblasts L929 were inoculated and cultured on the surface of SF microspheres. Observations by SEM and CLSM, as well as the results of cell counts and cell viability, showed that bFGF-loaded SF microspheres significantly promoted cell proliferation compared to bFGF-unloaded ones after 5 to 9 days of culture. This demonstrated the bFGF released from SF microspheres had significantly biological activity to promote cell growth. SF microspheres sustainably releasing bioactive bFGF might provide a new biocompatible matrix for the application to massive cell amplification and tissue engineering.
